# (Biphenyl-4-yl)[2-(4-methyl­benzo­yl)phen­yl]methanone

**DOI:** 10.1107/S1600536811047131

**Published:** 2011-11-12

**Authors:** V. Silambarasan, T. Srinivasan, S. Sivasakthikumaran, A. K. Mohanakrishnan, D. Velmurugan

**Affiliations:** a Centre of Advanced Study in Crystallography and Biophysics, University of Madras, Guindy Campus, Chennai 600 025, India; b Department of Organic Chemistry, University of Madras, Guindy Campus, Chennai 600 025, India

## Abstract

In the title compound, C_27_H_20_O_2_, the central benzene ring makes dihedral angles of 64.86 (7) and 70.35 (7)° with the methyl-substituted ring and the biphenyl ring system, respectively. The crystal packing is stabilized by inter­molecular C—H⋯O inter­actions, which link the mol­ecules into chains parallel to the *b* axis.

## Related literature

For the uses and biological importance of diketones, see: Bennett *et al.* (1999 ▶); Sato *et al.* (2008 ▶). For applications of biphenyl derivatives, see: Kucybala & Wrzyszczynski (2002 ▶). For related structures, see: Narayanan *et al.* (2011 ▶); Saeed *et al.* (2010 ▶).
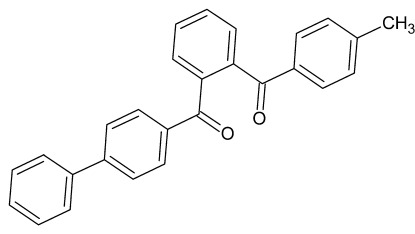


## Experimental

### 

#### Crystal data

C_27_H_20_O_2_
*M* *_r_* = 376.43
Monoclinic, 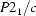

*a* = 22.2591 (5) Å
*b* = 7.7624 (2) Å
*c* = 11.4312 (2) Å
β = 97.454 (1)°
*V* = 1958.44 (8) Å^3^
*Z* = 4
Mo *K*α radiation
μ = 0.08 mm^−1^
*T* = 293 K
0.20 × 0.20 × 0.20 mm

#### Data collection

Bruker SMART APEXII area-detector diffractometer
18479 measured reflections
4860 independent reflections
3695 reflections with *I* > 2σ(*I*)
*R*_int_ = 0.032

#### Refinement

*R*[*F* ^2^ > 2σ(*F* ^2^)] = 0.047
*wR*(*F* ^2^) = 0.147
*S* = 1.01
4860 reflections
264 parameters
H-atom parameters constrained
Δρ_max_ = 0.26 e Å^−3^
Δρ_min_ = −0.20 e Å^−3^

### 

Data collection: *APEX2* (Bruker, 2008 ▶); cell refinement: *SAINT* (Bruker, 2008 ▶); data reduction: *SAINT*; program(s) used to solve structure: *SHELXS97* (Sheldrick, 2008 ▶); program(s) used to refine structure: *SHELXL97* (Sheldrick, 2008 ▶); molecular graphics: *ORTEP-3* (Farrugia, 1997 ▶); software used to prepare material for publication: *SHELXL97* and *PLATON* (Spek, 2009 ▶).

## Supplementary Material

Crystal structure: contains datablock(s) global, I. DOI: 10.1107/S1600536811047131/bt5706sup1.cif

Structure factors: contains datablock(s) I. DOI: 10.1107/S1600536811047131/bt5706Isup2.hkl

Supplementary material file. DOI: 10.1107/S1600536811047131/bt5706Isup3.cml

Additional supplementary materials:  crystallographic information; 3D view; checkCIF report

## Figures and Tables

**Table 1:** Hydrogen-bond geometry (Å, °)

*D*—H⋯*A*	*D*—H	H⋯*A*	*D*⋯*A*	*D*—H⋯*A*
C16—H16⋯01^i^	0.93	2.57	3.4196 (18)	152

Symmetry code: (i) 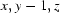.

**Fig. 1.**
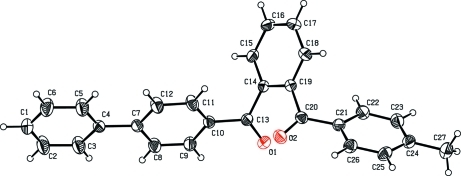
The molecular structure of the title compound, showing displacement ellipsoids drawn at the 30% probability level. H atoms are presented as a small spheres of arbitrary radius.

**Fig. 2.**
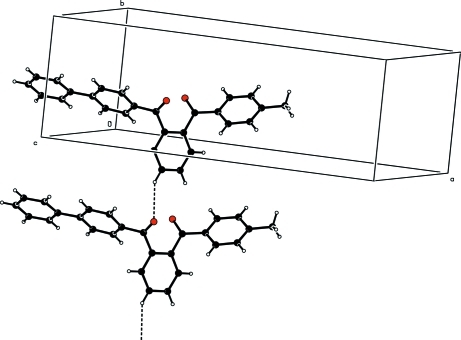
The crystal packing of the title compound viewed down b axis, showing the hydrogen bonds.

## supplementary crystallographic information

### Comment

Various biphenyl derivatives are used in the synthesis of pharmaceuticals, antifungal agents like bifonazole, optical brightening agents, dyes and polychlorinated biphenyls (PCBs). PCBs are used as heat-transfer agents, as electric insulators and are environmental pollutants causing carcinogenesis (Kucybala & Wrzyszczynski, 2002). Diketones are popular in organic synthesis for their applications in biology and medicine. They are known to exhibit antioxidants, antitumour and antibacterial activities (Bennett *et al.*,1999). They are also key intermediates in the preparation of various heterocyclic compounds (Sato *et al.*, 2008).

X-ray analysis confirms the molecular structure and atom connectivity of the title compound as illustrated in the Fig. 1. The central phenyl (C14–C19) ring makes dihedral angles of 64.86 (7)° and 70.35 (7)° with the methyl substituted phenyl ring (C21–C26) and the biphenyl ring system (C1–C12), respectively. The keto atoms O1 and O2 significantly deviate from the central phenyl ring (C14–C19) by –0.9393 (11)Å and 0.8857 (11)Å, respectively. The central phenyl ring makes dihedral angles of 57.16 (5)° and 47.51 (6)° with the ketone groups (C10/C13/C14/O1) and (C19/C20/C21/O2), respectively. The title compound exhibits structural similarities with the already reported related structure (Narayanan *et al.*, 2011).

The crystal packing is stabilized by C—H···O intermolecular interaction (Table 1). The C16—H16···O1^i^ interaction generates a C6 chain parallel to *b* axis (symmetry code: *x*, *y-1*, *z*). The packing of the title compound is shown in Fig. 2.

### Experimental

The furan (1 g) was dissolved in THF. The weighed lead tetracetone (1.52 g, 1520 mmol) was added to the furan. Then it was refluxed at 343 K for 0.5 h. The reaction mixture was analyzed by TLC. Then the usual workup was done with brine solution and CHCl_3_ followed by column chromatography (10% 10% AcOEt/hexane) which lead to the solution of the pure compound. Single crystals suitable for X–ray diffraction were obtained by slow evaporation of a solution of the title compound in ethyl acetate at room temperature.

### Refinement

The hydrogen atoms were placed in calculated positions with C—H = 0.93Å to 0.96Å and refined in the riding model with fixed isotropic displacement parameters: *U*_iso_(H) = 1.5*U*_eq_(C) for the methyl group and *U*_iso_(H) = 1.2*U*_eq_(C) for other H atoms.

### Crystal data

**Table:**

C_27_H_20_O_2_	*F*(000) = 792
*M**_r_* = 376.43	*D*_x_ = 1.277 Mg m^−^^3^
Monoclinic, *P*2_1_/*c*	Mo *K*α radiation, λ = 0.71073 Å
Hall symbol: -P 2ybc	Cell parameters from 4860 reflections
*a* = 22.2591 (5) Å	θ = 1.9–28.3°
*b* = 7.7624 (2) Å	µ = 0.08 mm^−^^1^
*c* = 11.4312 (2) Å	*T* = 293 K
β = 97.454 (1)°	Block, colourless
*V* = 1958.44 (8) Å^3^	0.20 × 0.20 × 0.20 mm
*Z* = 4	

### Data collection

**Table:**

Bruker SMART APEXII area-detector diffractometer	3695 reflections with *I* > 2σ(*I*)
Radiation source: fine-focus sealed tube	*R*_int_ = 0.032
graphite	θ_max_ = 28.3°, θ_min_ = 1.9°
ω and φ scans	*h* = −29→29
18479 measured reflections	*k* = −10→10
4860 independent reflections	*l* = −14→15

### Refinement

**Table:**

Refinement on *F*^2^	Secondary atom site location: difference Fourier map
Least-squares matrix: full	Hydrogen site location: inferred from neighbouring sites
*R*[*F*^2^ > 2σ(*F*^2^)] = 0.047	H-atom parameters constrained
*wR*(*F*^2^) = 0.147	*w* = 1/[σ^2^(*F*_o_^2^) + (0.0727*P*)^2^ + 0.5102*P*] where *P* = (*F*_o_^2^ + 2*F*_c_^2^)/3
*S* = 1.01	(Δ/σ)_max_ = 0.030
4860 reflections	Δρ_max_ = 0.26 e Å^−^^3^
264 parameters	Δρ_min_ = −0.20 e Å^−^^3^
0 restraints	Extinction correction: *SHELXL97* (Sheldrick, 2008), Fc^*^=kFc[1+0.001xFc^2^λ^3^/sin(2θ)]^-1/4^
Primary atom site location: structure-invariant direct methods	Extinction coefficient: 0.0107 (16)

### Special details

**Table:**

Geometry. All esds (except the esd in the dihedral angle between two l.s. planes) are estimated using the full covariance matrix. The cell esds are taken into account individually in the estimation of esds in distances, angles and torsion angles; correlations between esds in cell parameters are only used when they are defined by crystal symmetry. An approximate (isotropic) treatment of cell esds is used for estimating esds involving l.s. planes.
Refinement. Refinement of F^2^ against ALL reflections. The weighted R-factor wR and goodness of fit S are based on F^2^, conventional R-factors R are based on F, with F set to zero for negative F^2^. The threshold expression of F^2^ > 2sigma(F^2^) is used only for calculating R-factors(gt) etc. and is not relevant to the choice of reflections for refinement. R-factors based on F^2^ are statistically about twice as large as those based on F, and R- factors based on ALL data will be even larger.

### Fractional atomic coordinates and isotropic or equivalent isotropic displacement parameters (Å^2^)

**Table:**

	*x*	*y*	*z*	*U*_iso_*/*U*_eq_	
O1	0.28534 (5)	0.38124 (13)	0.65595 (10)	0.0506 (3)	
C13	0.24874 (6)	0.27565 (17)	0.61339 (11)	0.0377 (3)	
O2	0.28106 (5)	0.38387 (14)	0.38609 (10)	0.0544 (3)	
C14	0.26900 (5)	0.11095 (16)	0.56107 (12)	0.0375 (3)	
C7	0.05660 (6)	0.33333 (17)	0.61684 (12)	0.0392 (3)	
C20	0.32077 (6)	0.27867 (17)	0.41439 (12)	0.0409 (3)	
C21	0.38428 (6)	0.30892 (17)	0.39267 (12)	0.0405 (3)	
C10	0.18236 (6)	0.29702 (17)	0.61430 (11)	0.0386 (3)	
C19	0.30593 (6)	0.11233 (17)	0.47073 (13)	0.0410 (3)	
C4	−0.01000 (6)	0.34472 (17)	0.62034 (12)	0.0404 (3)	
C18	0.32434 (7)	−0.0436 (2)	0.42671 (16)	0.0544 (4)	
H18	0.3481	−0.0438	0.3655	0.065*	
C11	0.14137 (7)	0.2168 (2)	0.53081 (13)	0.0527 (4)	
H11	0.1554	0.1489	0.4731	0.063*	
C9	0.16008 (7)	0.4010 (2)	0.69754 (14)	0.0507 (4)	
H9	0.1868	0.4602	0.7526	0.061*	
C15	0.25338 (6)	−0.04573 (18)	0.60771 (14)	0.0472 (3)	
H15	0.2293	−0.0472	0.6684	0.057*	
C25	0.45437 (7)	0.4572 (2)	0.28431 (14)	0.0530 (4)	
H25	0.4610	0.5322	0.2238	0.064*	
C22	0.43345 (7)	0.2315 (2)	0.46015 (13)	0.0482 (3)	
H22	0.4268	0.1541	0.5192	0.058*	
C23	0.49216 (7)	0.2685 (2)	0.44031 (15)	0.0531 (4)	
H23	0.5245	0.2170	0.4873	0.064*	
C17	0.30771 (8)	−0.1976 (2)	0.47294 (18)	0.0609 (5)	
H17	0.3198	−0.3009	0.4421	0.073*	
C8	0.09836 (7)	0.4172 (2)	0.69900 (14)	0.0536 (4)	
H8	0.0843	0.4860	0.7563	0.064*	
C26	0.39563 (7)	0.4241 (2)	0.30508 (13)	0.0485 (3)	
H26	0.3635	0.4793	0.2601	0.058*	
C24	0.50361 (7)	0.3812 (2)	0.35152 (14)	0.0497 (4)	
C12	0.07980 (7)	0.2361 (2)	0.53183 (14)	0.0544 (4)	
H12	0.0532	0.1822	0.4737	0.065*	
C16	0.27311 (7)	−0.19908 (19)	0.56502 (17)	0.0573 (4)	
H16	0.2632	−0.3031	0.5980	0.069*	
C5	−0.05078 (8)	0.2644 (3)	0.53690 (18)	0.0706 (5)	
H5	−0.0363	0.2027	0.4766	0.085*	
C3	−0.03413 (8)	0.4333 (3)	0.70758 (17)	0.0674 (5)	
H3	−0.0082	0.4883	0.7663	0.081*	
C1	−0.13564 (7)	0.3628 (2)	0.62571 (17)	0.0599 (4)	
H1	−0.1773	0.3700	0.6269	0.072*	
C2	−0.09621 (8)	0.4423 (3)	0.7099 (2)	0.0786 (6)	
H2	−0.1112	0.5036	0.7698	0.094*	
C27	0.56713 (8)	0.4206 (3)	0.3272 (2)	0.0718 (5)	
H27A	0.5789	0.3394	0.2711	0.108*	
H27B	0.5944	0.4126	0.3993	0.108*	
H27C	0.5685	0.5351	0.2958	0.108*	
C6	−0.11250 (8)	0.2728 (3)	0.54021 (19)	0.0753 (6)	
H6	−0.1386	0.2159	0.4828	0.090*	

### Atomic displacement parameters (Å^2^)

**Table:**

	*U*^11^	*U*^22^	*U*^33^	*U*^12^	*U*^13^	*U*^23^
O1	0.0451 (6)	0.0452 (6)	0.0626 (7)	−0.0084 (4)	0.0114 (5)	−0.0064 (5)
C13	0.0380 (6)	0.0378 (7)	0.0384 (6)	−0.0015 (5)	0.0089 (5)	0.0035 (5)
O2	0.0517 (6)	0.0479 (6)	0.0665 (7)	0.0115 (5)	0.0188 (5)	0.0113 (5)
C14	0.0300 (6)	0.0359 (6)	0.0466 (7)	−0.0011 (5)	0.0052 (5)	0.0024 (5)
C7	0.0373 (6)	0.0397 (7)	0.0413 (7)	0.0034 (5)	0.0073 (5)	0.0015 (5)
C20	0.0451 (7)	0.0375 (7)	0.0421 (7)	0.0028 (6)	0.0133 (5)	−0.0010 (5)
C21	0.0432 (7)	0.0384 (7)	0.0417 (7)	−0.0007 (6)	0.0122 (5)	−0.0009 (5)
C10	0.0369 (6)	0.0393 (7)	0.0405 (6)	0.0019 (5)	0.0087 (5)	0.0024 (5)
C19	0.0366 (6)	0.0360 (7)	0.0517 (8)	0.0013 (5)	0.0108 (6)	0.0005 (5)
C4	0.0383 (7)	0.0379 (7)	0.0456 (7)	0.0037 (5)	0.0074 (5)	0.0027 (5)
C18	0.0524 (9)	0.0432 (8)	0.0707 (10)	0.0056 (7)	0.0201 (7)	−0.0062 (7)
C11	0.0422 (7)	0.0691 (10)	0.0469 (8)	0.0090 (7)	0.0060 (6)	−0.0192 (7)
C9	0.0409 (7)	0.0565 (9)	0.0555 (8)	−0.0048 (6)	0.0091 (6)	−0.0179 (7)
C15	0.0370 (7)	0.0426 (8)	0.0625 (9)	−0.0052 (6)	0.0077 (6)	0.0080 (6)
C25	0.0565 (9)	0.0518 (9)	0.0531 (8)	−0.0110 (7)	0.0160 (7)	0.0069 (7)
C22	0.0509 (8)	0.0487 (8)	0.0464 (7)	0.0034 (6)	0.0116 (6)	0.0068 (6)
C23	0.0450 (8)	0.0567 (9)	0.0574 (9)	0.0049 (7)	0.0057 (6)	−0.0004 (7)
C17	0.0544 (9)	0.0353 (8)	0.0933 (13)	0.0058 (7)	0.0102 (9)	−0.0072 (8)
C8	0.0440 (8)	0.0611 (9)	0.0576 (9)	−0.0001 (7)	0.0135 (7)	−0.0221 (7)
C26	0.0485 (8)	0.0493 (8)	0.0478 (8)	−0.0024 (6)	0.0067 (6)	0.0075 (6)
C24	0.0465 (8)	0.0480 (8)	0.0568 (9)	−0.0062 (6)	0.0151 (6)	−0.0105 (7)
C12	0.0401 (7)	0.0729 (11)	0.0483 (8)	0.0061 (7)	−0.0011 (6)	−0.0209 (7)
C16	0.0465 (8)	0.0344 (7)	0.0893 (12)	−0.0049 (6)	0.0021 (8)	0.0105 (7)
C5	0.0442 (9)	0.0904 (14)	0.0785 (12)	−0.0044 (9)	0.0123 (8)	−0.0382 (11)
C3	0.0443 (8)	0.0872 (13)	0.0711 (11)	0.0035 (8)	0.0091 (8)	−0.0318 (10)
C1	0.0381 (7)	0.0653 (10)	0.0778 (11)	0.0021 (7)	0.0130 (7)	0.0006 (9)
C2	0.0476 (9)	0.0999 (16)	0.0911 (14)	0.0064 (10)	0.0200 (9)	−0.0371 (12)
C27	0.0503 (9)	0.0731 (12)	0.0956 (14)	−0.0116 (9)	0.0233 (9)	−0.0098 (11)
C6	0.0417 (9)	0.0968 (15)	0.0863 (13)	−0.0110 (9)	0.0047 (8)	−0.0311 (12)

### Geometric parameters (Å, °)

**Table:**

O1—C13	1.2122 (16)	C25—C26	1.383 (2)
C13—C10	1.4881 (18)	C25—C24	1.386 (2)
C13—C14	1.5052 (18)	C25—H25	0.9300
O2—C20	1.2159 (17)	C22—C23	1.385 (2)
C14—C15	1.3901 (18)	C22—H22	0.9300
C14—C19	1.4009 (19)	C23—C24	1.388 (2)
C7—C12	1.3825 (19)	C23—H23	0.9300
C7—C8	1.394 (2)	C17—C16	1.383 (3)
C7—C4	1.4908 (18)	C17—H17	0.9300
C20—C21	1.4854 (19)	C8—H8	0.9300
C20—C19	1.4987 (19)	C26—H26	0.9300
C21—C26	1.3897 (19)	C24—C27	1.507 (2)
C21—C22	1.391 (2)	C12—H12	0.9300
C10—C11	1.380 (2)	C16—H16	0.9300
C10—C9	1.3878 (19)	C5—C6	1.381 (2)
C19—C18	1.3929 (19)	C5—H5	0.9300
C4—C3	1.376 (2)	C3—C2	1.387 (2)
C4—C5	1.378 (2)	C3—H3	0.9300
C18—C17	1.377 (2)	C1—C6	1.356 (3)
C18—H18	0.9300	C1—C2	1.363 (3)
C11—C12	1.380 (2)	C1—H1	0.9300
C11—H11	0.9300	C2—H2	0.9300
C9—C8	1.382 (2)	C27—H27A	0.9600
C9—H9	0.9300	C27—H27B	0.9600
C15—C16	1.380 (2)	C27—H27C	0.9600
C15—H15	0.9300	C6—H6	0.9300
			
O1—C13—C10	122.55 (12)	C22—C23—C24	121.14 (15)
O1—C13—C14	120.89 (12)	C22—C23—H23	119.4
C10—C13—C14	116.52 (11)	C24—C23—H23	119.4
C15—C14—C19	119.29 (12)	C18—C17—C16	120.25 (14)
C15—C14—C13	119.20 (12)	C18—C17—H17	119.9
C19—C14—C13	121.41 (11)	C16—C17—H17	119.9
C12—C7—C8	116.82 (12)	C9—C8—C7	121.77 (13)
C12—C7—C4	120.98 (12)	C9—C8—H8	119.1
C8—C7—C4	122.19 (12)	C7—C8—H8	119.1
O2—C20—C21	121.61 (13)	C25—C26—C21	120.51 (14)
O2—C20—C19	119.99 (12)	C25—C26—H26	119.7
C21—C20—C19	118.39 (12)	C21—C26—H26	119.7
C26—C21—C22	118.29 (13)	C25—C24—C23	117.80 (14)
C26—C21—C20	119.21 (13)	C25—C24—C27	120.31 (16)
C22—C21—C20	122.44 (12)	C23—C24—C27	121.89 (16)
C11—C10—C9	118.26 (12)	C11—C12—C7	121.81 (13)
C11—C10—C13	120.85 (12)	C11—C12—H12	119.1
C9—C10—C13	120.87 (12)	C7—C12—H12	119.1
C18—C19—C14	119.20 (13)	C15—C16—C17	119.75 (14)
C18—C19—C20	120.14 (12)	C15—C16—H16	120.1
C14—C19—C20	120.42 (11)	C17—C16—H16	120.1
C3—C4—C5	116.35 (14)	C4—C5—C6	121.87 (16)
C3—C4—C7	122.13 (13)	C4—C5—H5	119.1
C5—C4—C7	121.51 (13)	C6—C5—H5	119.1
C17—C18—C19	120.63 (15)	C4—C3—C2	121.48 (16)
C17—C18—H18	119.7	C4—C3—H3	119.3
C19—C18—H18	119.7	C2—C3—H3	119.3
C12—C11—C10	120.89 (13)	C6—C1—C2	118.16 (15)
C12—C11—H11	119.6	C6—C1—H1	120.9
C10—C11—H11	119.6	C2—C1—H1	120.9
C8—C9—C10	120.36 (13)	C1—C2—C3	121.03 (17)
C8—C9—H9	119.8	C1—C2—H2	119.5
C10—C9—H9	119.8	C3—C2—H2	119.5
C16—C15—C14	120.81 (14)	C24—C27—H27A	109.5
C16—C15—H15	119.6	C24—C27—H27B	109.5
C14—C15—H15	119.6	H27A—C27—H27B	109.5
C26—C25—C24	121.53 (14)	C24—C27—H27C	109.5
C26—C25—H25	119.2	H27A—C27—H27C	109.5
C24—C25—H25	119.2	H27B—C27—H27C	109.5
C23—C22—C21	120.71 (14)	C1—C6—C5	121.10 (17)
C23—C22—H22	119.6	C1—C6—H6	119.4
C21—C22—H22	119.6	C5—C6—H6	119.4
			
O1—C13—C14—C15	120.10 (15)	C19—C14—C15—C16	−0.9 (2)
C10—C13—C14—C15	−57.39 (16)	C13—C14—C15—C16	−177.50 (13)
O1—C13—C14—C19	−56.44 (18)	C26—C21—C22—C23	0.0 (2)
C10—C13—C14—C19	126.07 (13)	C20—C21—C22—C23	−177.29 (14)
O2—C20—C21—C26	−22.7 (2)	C21—C22—C23—C24	−1.1 (2)
C19—C20—C21—C26	156.20 (13)	C19—C18—C17—C16	−0.9 (3)
O2—C20—C21—C22	154.55 (15)	C10—C9—C8—C7	−1.1 (3)
C19—C20—C21—C22	−26.53 (19)	C12—C7—C8—C9	−1.5 (2)
O1—C13—C10—C11	155.07 (15)	C4—C7—C8—C9	177.69 (15)
C14—C13—C10—C11	−27.48 (19)	C24—C25—C26—C21	−1.8 (2)
O1—C13—C10—C9	−23.5 (2)	C22—C21—C26—C25	1.4 (2)
C14—C13—C10—C9	153.91 (13)	C20—C21—C26—C25	178.82 (13)
C15—C14—C19—C18	2.3 (2)	C26—C25—C24—C23	0.7 (2)
C13—C14—C19—C18	178.87 (13)	C26—C25—C24—C27	−179.65 (16)
C15—C14—C19—C20	176.73 (12)	C22—C23—C24—C25	0.8 (2)
C13—C14—C19—C20	−6.7 (2)	C22—C23—C24—C27	−178.89 (15)
O2—C20—C19—C18	129.29 (16)	C10—C11—C12—C7	−1.1 (3)
C21—C20—C19—C18	−49.65 (19)	C8—C7—C12—C11	2.6 (3)
O2—C20—C19—C14	−45.1 (2)	C4—C7—C12—C11	−176.60 (15)
C21—C20—C19—C14	136.01 (13)	C14—C15—C16—C17	−1.5 (2)
C12—C7—C4—C3	176.57 (17)	C18—C17—C16—C15	2.4 (3)
C8—C7—C4—C3	−2.6 (2)	C3—C4—C5—C6	0.3 (3)
C12—C7—C4—C5	−2.4 (2)	C7—C4—C5—C6	179.32 (18)
C8—C7—C4—C5	178.45 (17)	C5—C4—C3—C2	−0.8 (3)
C14—C19—C18—C17	−1.5 (2)	C7—C4—C3—C2	−179.81 (18)
C20—C19—C18—C17	−175.88 (15)	C6—C1—C2—C3	0.7 (3)
C9—C10—C11—C12	−1.6 (2)	C4—C3—C2—C1	0.3 (4)
C13—C10—C11—C12	179.78 (15)	C2—C1—C6—C5	−1.2 (3)
C11—C10—C9—C8	2.6 (2)	C4—C5—C6—C1	0.7 (4)
C13—C10—C9—C8	−178.71 (14)		

### Hydrogen-bond geometry (Å, °)

**Table:**

*D*—H···*A*	*D*—H	H···*A*	*D*···*A*	*D*—H···*A*
C16—H16···01i	0.93	2.57	3.4196 (18)	152

Symmetry codes: i.
